# CUBAP: an interactive web portal for analyzing codon usage biases across populations

**DOI:** 10.1093/nar/gkaa863

**Published:** 2020-10-12

**Authors:** Matthew W Hodgman, Justin B Miller, Taylor E Meurs, John S K Kauwe

**Affiliations:** Department of Biology, Brigham Young University, Provo, UT 84602, USA; Department of Biology, Brigham Young University, Provo, UT 84602, USA; Department of Biology, Brigham Young University, Provo, UT 84602, USA; Department of Biology, Brigham Young University, Provo, UT 84602, USA

## Abstract

Synonymous codon usage significantly impacts translational and transcriptional efficiency, gene expression, the secondary structure of both mRNA and proteins, and has been implicated in various diseases. However, population-specific differences in codon usage biases remain largely unexplored. Here, we present a web server, https://cubap.byu.edu, to facilitate analyses of codon usage biases across populations (CUBAP). Using the 1000 Genomes Project, we calculated and visually depict population-specific differences in codon frequencies, codon aversion, identical codon pairing, co-tRNA codon pairing, ramp sequences, and nucleotide composition in 17,634 genes. We found that codon pairing significantly differs between populations in 35.8% of genes, allowing us to successfully predict the place of origin for African and East Asian individuals with 98.8% and 100% accuracy, respectively. We also used CUBAP to identify a significant bias toward decreased CTG pairing in the immunity related GTPase M (*IRGM*) gene in East Asian and African populations, which may contribute to the decreased association of *rs10065172* with Crohn's disease in those populations. CUBAP facilitates in-depth gene-specific and codon-specific visualization that will aid in analyzing candidate genes identified in genome-wide association studies, identifying functional implications of synonymous variants, predicting population-specific impacts of synonymous variants and categorizing genetic biases unique to certain populations.

## INTRODUCTION

Ribosomes translate 61 unique codons into 20 amino acids because a natural wobble allows aminoacyl-tRNA to bind one of several different codons through non-Watson-Crick pairing that typically occurs at the third nucleotide position (1,2). Codon usage biases arise when synonymous codons occur in different frequencies across genomes, genes, or positions within genes. Codon usage biases affect translation efficiency (3–5), gene expression levels (6–9) and mRNA and protein secondary structure (10–17). Codon optimality and codon frequency directly correlate with tRNA abundance (6,18–20), and more abundant codons are generally translated faster, which increases overall protein levels (6,9,21,22). Codons with rare cognate tRNAs are translated slower than optimal codons and generally decrease gene expression because of limited cognate tRNA availability, which increases tRNA competition at that codon position (21,22). Therefore, codon usage biases that favor optimal codons can lead to complete aversion to suboptimal codons, which in turn decreases resource utilization, increases translational speed, and usually increases gene expression (6,10,21,23,24). Additionally, codon choice can alter mRNA and protein secondary structure through hydrogen bonding and co-translational folding, which also affects transcriptional and translational speed (10–17).

Recent studies show that specific locations within genes are also susceptible to codon usage biases for either optimal or suboptimal codons (3,4,11,25,26). Identical codon pairing occurs when a codon appears multiple times within a single ribosomal reading frame. Similarly, co-tRNA codon pairing occurs when multiple synonymous, but not identical, codons occur within a single reading frame. These pairings allow ribosomes to recharge tRNAs before they diffuse from the ribosome, effectively increasing translation speed and gene expression (3,11,25). A small ramp sequence of suboptimal, slowly-translated codons at the beginning of a gene surprisingly increases translational efficiency (26) by preventing downstream ribosomal ‘traffic jams’ (5,27). Additionally, ramp sequences coincide with more efficient hydrogen bonding that increases transcriptional efficiency by requiring fewer dsDNA bonds to be broken (28). More information on codon pairing and ramp sequences, including an example of each, are depicted in Supplementary Figures S1 and S2.

Since codon usage biases directly affect protein levels and protein structure, they likely have a non-trivial effect on genetic diseases and disorders. Synonymous mutations have been associated with >30 different diseases (29). In some cases, the etiological effects are attributed to the synonymous mutation altering splicing (30–34), although in many cases the biological mechanisms driving the synonymous variant association with disease remain unknown. For example, various synonymous mutations in the *MDR1* gene are associated with drug resistance for a variety of diseases including cancer (35–42). Additionally, a synonymous mutation in *UBE1* associated with developing X-linked infantile spinal muscular atrophy significantly lowers *UBE1* expression and changes exon methylation (43). Synonymous rare variants are also associated with Alzheimer's disease biomarkers (44), and shifts in codon usage biases alone can yield high diagnostic accuracy in a variety of other diseases including cancers and multiple sclerosis (45). Furthermore, codon usage biases in rat oncogenes directly regulate tumor growth (46). Therefore, better understanding the effects of codon usage on protein expression may lead to improved protein therapeutics and better understanding the etiology of various diseases (29).

The 1000 Genomes Project contains whole genome sequencing data from 2504 individuals spanning 26 populations from five superpopulations and has been widely used to assess population-specific genetic differences. Although specific genomic inquiries of the 1000 Genomes Project can be conducted on web portals such as PopHuman (47) and CAGm (48), population-specific differences in codon usage biases remain largely unexplored and are not easily accessible. Tools such as CAICal (49) and CodonW (50) specialize in gene-specific or individual-specific calculations of codon usage biases. Codon metrics such as the Codon Adaptation Index (51), Relative Synonymous Codon Usage (52), the Effective Number of Codons (53), and GC content are often used to compare codon usage biases between small groups of individuals or species. GC content is commonly used in studies of codon usage biases because higher GC content is indicative of genic regions (54,55) and correlates with the overall nucleotide composition of the genome (56). Some databases, such as HIVE-CUT (57) and CBDB (58), enable phylogenic comparisons of species-specific codon usage biases. While these methods allow researchers to compare codon usage statistics between studies and species, the extent to which differences in specific codon usage biases (e.g. ramp sequences, codon pairing (59), codon aversion (60)) differ between human populations and their ability to predict population of origin have yet to be characterized. Additionally, a centralized database that can be used to identify conserved population-specific differences in codon usage biases that might have disease implications does not currently exist. Because codon optimality correlates with codon frequency, the effects of a synonymous mutations on protein secondary structure, expression, and disease may vary between populations where population-specific codon biases exist. A database of population-specific codon usage biases would provide an additional filter for evaluating findings from genome-wide association studies, elucidate the extent to which synonymous variants affect disease susceptibility in different populations, and provide insights into population-specific reactions to drugs in clinical trials.

Our web portal, Codon Usage Biases Across Populations (https://cubap.byu.edu), facilitates these types of analyses by providing users with annotated codon usage biases across different human populations using data from the 1000 Genomes Project. We used these biases to accurately predict the population of origin in African and East Asian populations with 98.8% and 100% accuracy, respectively. Additionally, CUBAP’s dataset can be used to better understand how synonymous variants may affect disease development in a variety of ways, and we provide a case study of how CUBAP can be used to analyze the effects of pathogenic synonymous variants. CUBAP includes various analyses of codon frequencies, codon aversion, identical codon pairing, co-tRNA codon pairing, ramp sequences, and nucleotide composition including GC content spanning 17 634 genes and 40 643 isoforms. Interactive graphics powered by Microsoft Power BI facilitate custom project-specific analyses of codon usages within specific populations, genes, or codons.

## MATERIALS AND METHODS

### Data availability

All analyses were performed on 2504 human samples from the 1000 Genomes Project database. This dataset was selected because of its credibility, accuracy, and large number of samples spanning many different populations. Individuals are labeled as belonging to one of 26 different subpopulations spanning five superpopulations (see Table 1). After excluding genes that had annotated translational exceptions, partial genes, or potential errors, we analyzed 17 634 complete gene sequences and 40 643 isoforms using human reference assembly hg19. All scripts used to analyze these data are publicly available at https://github.com/kauwelab/cubap. CUBAP’s documentation can be viewed at https://cubap.readthedocs.io/.

**Table 1.** Population data used from 1000 Genomes Project

Superpopulation	Subpopulation	Description	Number of Samples
Africa	ASW	African Ancestry in Southwest US	61
	ACB	African Caribbean in Barbados	96
	ESN	Esan in Nigeria	99
	GWD	Gambian in Western Division, The Gambia	113
	LWK	Luhya in Webuye, Kenya	99
	MSL	Mende in Sierra Leone	85
	YRI	Yoruba in Ibadan, Nigeria	108
		**Total:**	**661**
America	CLM	Colombian in Medellin, Colombia	94
	MXL	Mexican Ancestry in Los Angeles, California	64
	PEL	Peruvian in Lima, Peru	85
	PUR	Puerto Rican in Puerto Rico	104
		**Total:**	**347**
East Asia	CDX	Chinese Dai in Xishuangbanna, China	93
	CHB	Han Chinese in Bejing, China	103
	JPT	Japanese in Tokyo, Japan	104
	KHV	Kinh in Ho Chi Minh City, Vietnam	99
	CHS	Southern Han Chinese, China	105
		**Total:**	**504**
Europe	GBR	British in England and Scotland	91
	FIN	Finnish in Finland	99
	IBS	Iberian populations in Spain	107
	TSI	Toscani in Italy	107
	CEU	Utah residents with Northern and Western European ancestry	99
		**Total:**	**503**
South Asia	BEB	Bengali in Bangladesh	86
	GIH	Gujarati Indian in Houston, TX	103
	ITU	Indian Telugu in the UK	102
	PJL	Punjabi in Lahore, Pakistan	96
	STU	Sri Lankan Tamil in the UK	102
		**Total:**	**489**
		**Grand Total:**	**2,504**

The distribution of samples and populations within the 1000 Genomes Project.

### Calculating codon usage biases

We calculated various codon usage biases for each individual and population in our dataset. Codon frequency biases were calculated for each person by incrementing a counter for each codon and writing the resulting values for each gene to a comma separated values (CSV) file. Using the codon frequency CSV file, we calculated codon aversion data for each gene by isolating only codons that had a frequency of zero in any given gene or isoform.

We calculated identical and co-tRNA codon pairing frequencies for each gene using a modified script from Miller *et al.* (59). Identical codon pairing was calculated for each of the 61 amino-acid encoding codons by counting the number of times the same codon occurred within a ribosomal window of nine codons, which encompasses the average length of a ribosome (61). Similarly, co-tRNA codon pairing was calculated for each of 20 amino acids by counting the number of times non-identical codons that encode the same amino acid occurred multiple times within a ribosomal window. The resulting identical codon pairing and co-tRNA codon pairing data were also written to separate CSV files that can be downloaded from the website.

We used ExtRamp (27) to calculate the relative synonymous codon usage of each codon within each gene, and we determined the length of a ramp sequence (if it existed) within that gene for an individual. A ramp sequence is a short segment of suboptimal, slowly-translated codons at the beginning of a gene that is predicted to increase gene expression by preventing downstream ribosome collisions. ExtRamp (27) uses the relative synonymous codon usages calculated from all longest isoforms in human reference assembly hg19. Those relative synonymous codon usage values are used to calculate the harmonic mean translational speed within a sliding ribosomal window (e.g. nine codons), calculated for all ribosomal windows across a gene sequence. If an outlier local minimum occurs within the first percentile of the gene (i.e. the beginning of the gene), then a ramp sequence is written to an output file including the nucleotides at the beginning of the gene sequence through the end of the outlier region. We computed and report the harmonic mean of the relative synonymous codon usages in the ramp sequence as well as the harmonic mean of all codons within the gene sequence for each gene containing a predicted ramp sequence. We used the harmonic mean instead of the arithmetic or geometric mean because it is most appropriate for averaging ratios such as the relative synonymous codon usage, it is not significantly affected by outliers that may exist within the ramp sequence, and it was used by ExtRamp to calculate the existence of a ramp sequence (27). Results were written to a CSV file that can also be downloaded from https://cubap.byu.edu.

Nucleotide frequencies and GC content were also calculated by counting the occurrences of each nucleotide in each gene. GC content was computed for each gene within Microsoft Power BI.

Supplementary Figure S3 depicts how data were generated for the CUBAP server and details the format of the CSVs of each codon usage bias file.

### Population identification

#### Population-specific differences in codon usage biases

We performed an analysis of variance (ANOVA) on population-specific differences in codon frequency, co-tRNA codon pairs, identical codon pairs, codon aversion, and ramp sequences across the longest isoforms of 17 634 genes. The purpose of an ANOVA is to determine the significance of a relationship between a numeric variable (e.g. mean number of codon pairings) and categorical groups (e.g. population). Since codon frequencies, codon aversion, codon pairing, and ramp sequences were discrete analyses, a Bonferroni significance threshold was established for each of those biases independently depending on the number of genes and codons analyzed for each bias (see Supplementary Table S1). Pairwise t-tests were performed on all significant ANOVAs to determine which populations significantly differed from each other. We established a Bonferroni significance threshold for each set of t-tests by using the total number of tests for each codon bias (see Supplementary Table S1). Additionally, we calculated the Cohen's d (62) effect size for all significant t-tests. The ANOVA and t-tests are well-justified for eliciting significant population-specific differences in codon usages because the superpopulations and subpopulations were well-represented and consisted of large sample sizes (see Table 1).

Since codon aversion and codon pairing have previously been used to recover phylogenies, we also performed a single one-way ANOVA on the overall codon pairing frequencies of the five superpopulations and a second ANOVA on the overall codon aversion frequencies between each superpopulation to determine if overall codon pairing or codon aversion biases might be used in an alignment-free clustering algorithm to predict the population of origin of each individual in the 1000 Genomes Project. Since both ANOVAs were significant (*P*-values = 2.56 × 10^−189^ and 5.03 × 10^−31^, respectively), we proceeded to cluster individuals in the 1000 Genomes Project based on codon pairing or codon aversion across the genome.

#### Clustering

We assessed the predictive power of codon usage biases at identifying the superpopulation of origin for each person in the 1000 Genomes Project, as well as the hg19 human reference genome, by using two alignment-free phylogenomic algorithms to classify individuals based on codon aversion (60) and codon pairing (59). These phylogenomic algorithms perform similar calculations by first calculating a motif of codons that are either averted or pair at least once within a single gene. This process is repeated for all genes, and each motif is added to a set for a genome. Next, a distance is calculated between genomes using a set union of the motifs between individuals, without regard to gene names. Finally, a phylogeny is recovered using neighbor-joining. These phylogenies were used as pedigrees to assess the effectiveness of population clustering.

#### Evaluation of population identification

After recovering the proposed phylogeny using only codon pairing or codon aversion, we counted the unique clusters (i.e. clades) of individuals originating from the same superpopulation using the 1000 Genomes Project population annotations. Finally, we calculated the percent predictive accuracy for each of the five superpopulations as follows, where a cluster was defined as the largest discrete grouping that consisted of only individuals from the same superpopulation:

$$\begin{eqnarray*} &&{\rm{Percent}}\ {\rm{Predictive\ Accuracy\ }} \nonumber \\ && = 100\left( {1 - \frac{{{\rm{number\ of\ clusters}} - 1}}{{{\rm{number\ of\ individuals}}}}} \right) \end{eqnarray*}$$

The scripts used to perform these calculations are available at https://github.com/kauwelab/cubap/population_stratification.

### Data analysis & visualization

We opted to use Microsoft Power BI (Microsoft, 2019, Power BI Desktop) to visualize codon usage biases on a server (https://cubap.byu.edu) because of its rich library of interactive graphics that allow for optimized, state-of-the-art subsetting and visualization. Power BI allows users to interact with bar charts, violin plots, and box plots for each of the codon usage biases, genes, and populations. However, Power BI does not allow users to upload their own data to reports published on websites. Since the purpose of CUBAP is to provide a tool to query results from the 1000 Genomes Project, Power BI was optimally suited to allow user-specific queries of these data. We used the Power BI custom visual Violin Plot (Daniel Marsh-Patrick, version 1.3.0.4, 2019) that calculates the maximum, minimum, median, mean, and standard deviation for the number of codons or codon pairs for each population group. We used the Box and Whisker Chart custom visual (Jan Pieter Posthuma, version 2.5.3.0, 2017) to depict the relative synonymous codon usage for ramp sequences and the whole gene sequence. Users can interact with most of the visualizations on CUBAP by selecting specific genes, populations, and codons. We also implemented custom visual Smart Filters (OKViz, version 1.2.5.0, 2020) to improve the performance time of gene queries.

### Case study: evaluating population-specific biases in pathogenic synonymous variants

The extent to which population-specific codon usage biases affect disease susceptibility or synonymous variant pathogenicity within specific populations remains largely unknown. We mapped the minor allele frequencies (MAF) of 15 synonymous variants labeled ‘Pathogenic’ and supported by multiple submitters in ClinVar (62) across the 1000 Genomes Project to identify synonymous variants with a MAF that significantly differed between populations. Supplementary Table S2 shows the ANOVA *P*-values of comparing population-specific MAFs of these 15 synonymous pathogenic variants. We found that *rs10065172* (313C>T; p.Leu105Leu) in the immunity related GTPase M (*IRGM*) gene had a significantly different MAF between superpopulations (*P*-value = 1.06 × 10^−10^), after correcting for multiple tests using a Bonferroni correction (0.05/15 variants; α = 0.003333). By performing a Tukey test on *rs10065172* population-specific MAFs, we found that both Africa and East Asia were significantly different from all other superpopulations (*P*-values ≤ 6.096 × 10^−4^) except each other (*P*-value = 0.764) (see Supplementary Table S3 for complete comparisons). We then used CUBAP to identify population-specific codon usage biases (e.g., codon pairing, codon aversion, ramp sequences, and codon frequency) affecting *IRGM*, and evaluated the effects of *rs10065172* on changing these biases. We performed a literature search on *rs10065172* to identify population-specific differences in pathogenicity and compared those reported differences to the population-specific codon usage biases in *IRGM* reported by CUBAP.

## RESULTS

CUBAP features six interactive Power BI visuals for analyzing codon frequency, identical codon pairing, co-tRNA codon pairing, codon aversion, ramp sequences, and nucleotide composition (see Supplementary Figures S4–S9 for example screenshots). Apart from the ramp sequences visual, which includes only genes that have a ramp sequence, each of those visuals allows users to analyze codon usage biases for 40 643 isoforms spanning 17 634 genes in the human genome from all 2504 individuals encompassing 26 subpopulations and five superpopulations included in the 1000 Genomes Project. All raw CSV data files are available for download online by navigating to the ‘Results’ page of https://cubap.byu.edu.

Interactive bar charts for codon frequency, identical codon pairing, and co-tRNA codon pairing allow users to analyze these codon usage biases in detail (see Supplementary Figures S4-S6 for example screenshots). Each user query displays the mean and standard deviation for the user-selected gene or isoform and allows users to easily subset the results or query different genes. When analyzing multiple genes or isoforms, users can view codon usage data either as an average across all isoforms or for each isoform individually. Users can compare codon usage frequencies across all sub- and superpopulations using violin plots by selecting specific codons or amino acids.

Codon aversion plots allow users to analyze codon aversion frequencies in different genes, populations, and across the genome. Codon aversion is the absence of specific codons in a gene sequence. Often, averted codons correlate to rare tRNAs that are expected to slow translation. Supplementary Figure S7 shows an example of a codon aversion query on the website. Results from Power BI allow users to view and subset the total number of alleles in each subpopulation or superpopulation that use specific codons.

In the ramp sequence report, users can compare population frequencies of ramp sequences in specific genes (see Supplementary Figure S8 for an example screenshot). These sequences of slowly-translated codons at the beginning of a gene evenly space ribosomes to increase translation efficiency and can provide insight into gene and population-specific expression levels. Box and whisker plots show the harmonic mean of the relative synonymous codon usages of codons in both the ramp sequence and the entire gene. These data are then plotted across all subpopulations. Additionally, users may compare the average length of the ramp sequence to the length of the selected gene.

Users can view nucleotide frequencies and calculate GC content using the interactive visual for nucleotide composition (see Supplementary Figure S9). Interactive bar charts show the average frequency and standard deviation of each nucleotide for user-selected genes. Users can select a specific nucleotide to view population-specific frequencies on both bar and violin plots. Lines depicting mean GC content for the gene and across all populations are also plotted on the violin plot.

CUBAP is a resource for analyzing population differences in codon usage bias and does not allow users to upload data. However, users can calculate codon usage biases in their own data using the python scripts provided at https://github.com/kauwelab/cubap. These scripts require input data in a FASTA file format, which necessitates including both the reference sequence and the mutated sequence for variant analyses. These data can be compared directly to the 1000 Genomes Project codon usage bias data available at https://cubap.byu.edu/Results.html#data.

A single ANOVA test conducted on the total number of codon pairs in each genome significantly differed between superpopulations (*P*-value = 2.56 × 10^−189^). A second ANOVA test found that codon aversion also significantly differed between superpopulations (*P*-value = 5.03 × 10^−31^), indicating that these biases may be used as a metric to stratify populations. We performed 10 756 740 additional pairwise *t*-tests for each protein-encoding codon in each gene in each superpopulation, which resulted in 35.8% of all genes having at least one codon with a statistically significant difference in the average number of codon pairs between at least two superpopulations after correcting for multiple tests using a Bonferroni correction (*P*-value < 4.64 × 10^−9^). Aversion to any of the 64 codons, including the stop codons, significantly differed between at least two populations in at least one codon in 9.9% of genes after a Bonferroni correction (*P*-value < 4.43 × 10^−9^). The striking population differences in codon pairing and codon aversion between populations allowed us to use alignment-free phylogenetic algorithms to predict the superpopulation of origin for people in the 1000 Genomes Project. We also included the hg19 reference genome, which may have caused additional splits between clusters of individuals originating from the same population. Using codon pairing alone, we successfully identified the East Asian population (*n* = 504) with 100% accuracy and the African population (*n* = 661) with 98.8% accuracy. Table 2 shows the percent predictive accuracy for all populations. Codon aversion has similarly high predictive power within these populations, and accurately recovered 95.0% of the East Asian population and 85.9% of the African population (see Table 3 for all population comparisons). Although codon usage biases in American and European populations are difficult to differentiate, when stratified as a single group, 91.6% of individuals clustered in a single cluster using only codon pairing. Gene-specific analyses of the most statistically significant differences between populations are found at https://cubap.byu.edu/population_stratification.html.

**Table 2.** Population identification accuracy for codon pairing

Population	Total individuals	Number of clusters	Percent accuracy
**East Asia**	504	1	100
**Africa**	661	9	98.7897
**South Asia**	489	56	88.7526
**Europe**	503	91	82.1074
**America**	347	100	71.4697

Using an alignment-free phylogenomic algorithm that analyzes only codon pairing usages across a genome, individuals in the 1000 Genomes Projects were clustered in a phylogeny (i.e. similar to a pedigree). Cluster accuracy was determined based on the number of clusters (i.e. clades) of individuals belonging the same superpopulation, where a new cluster was formed when an individual from a different population was added to the cluster. The table shows the superpopulation, the number of individuals in that population, the number of clusters identified using the phylogenomic algorithm, and the percent accuracy of the classification.

**Table 3.** Population identification accuracy for codon aversion

Population	Total individuals	Number of clusters	Percent accuracy
**East Asia**	504	26	95.0397
**Africa**	661	94	85.9304
**Europe**	503	216	57.2565
**South Asia**	489	214	56.4417
**America**	347	165	52.7378

Using an alignment-free phylogenomic algorithm that analyzes only codon aversion usages across a genome, individuals in the 1000 Genomes Projects were clustered in a phylogeny (i.e. similar to a pedigree). Cluster accuracy was determined based on the number of clusters (i.e. clades) of individuals belonging the same superpopulation, where a new cluster was formed when an individual from a different population was added to the cluster. The table shows the superpopulation, the number of individuals in that population, the number of clusters identified using the phylogenomic algorithm and the percent accuracy of the classification.

### Case study: Crohn's disease and *rs10065172*

Several genome-wide association studies suggest *rs10065172* in the *IRGM* gene is strongly associated with Crohn's disease in European (odds ratio (OR) = 1.284; 95% confidence interval (CI) = 1.055–1.564) and Korean individuals (OR = 1.42; 95% CI = 1.12–1.80), but not in Japanese (OR = 0.94; 95% CI = 0.81–1.11) nor African American individuals (OR = 0.91 95% CI = 0.67–1.33) (63–69). When accounting for population stratification, the association between *rs10065172* and Crohn's disease increases in Europeans, but not Asians (69). Notably, Korean individuals are not included in the 1000 Genomes Project and thus not included in our analyses. While other variants are in near perfect linkage disequilibrium with *rs10065172*, *rs10065172* specifically increases Crohn's disease susceptibility in individuals of European ancestry (70).

**Figure 1.**
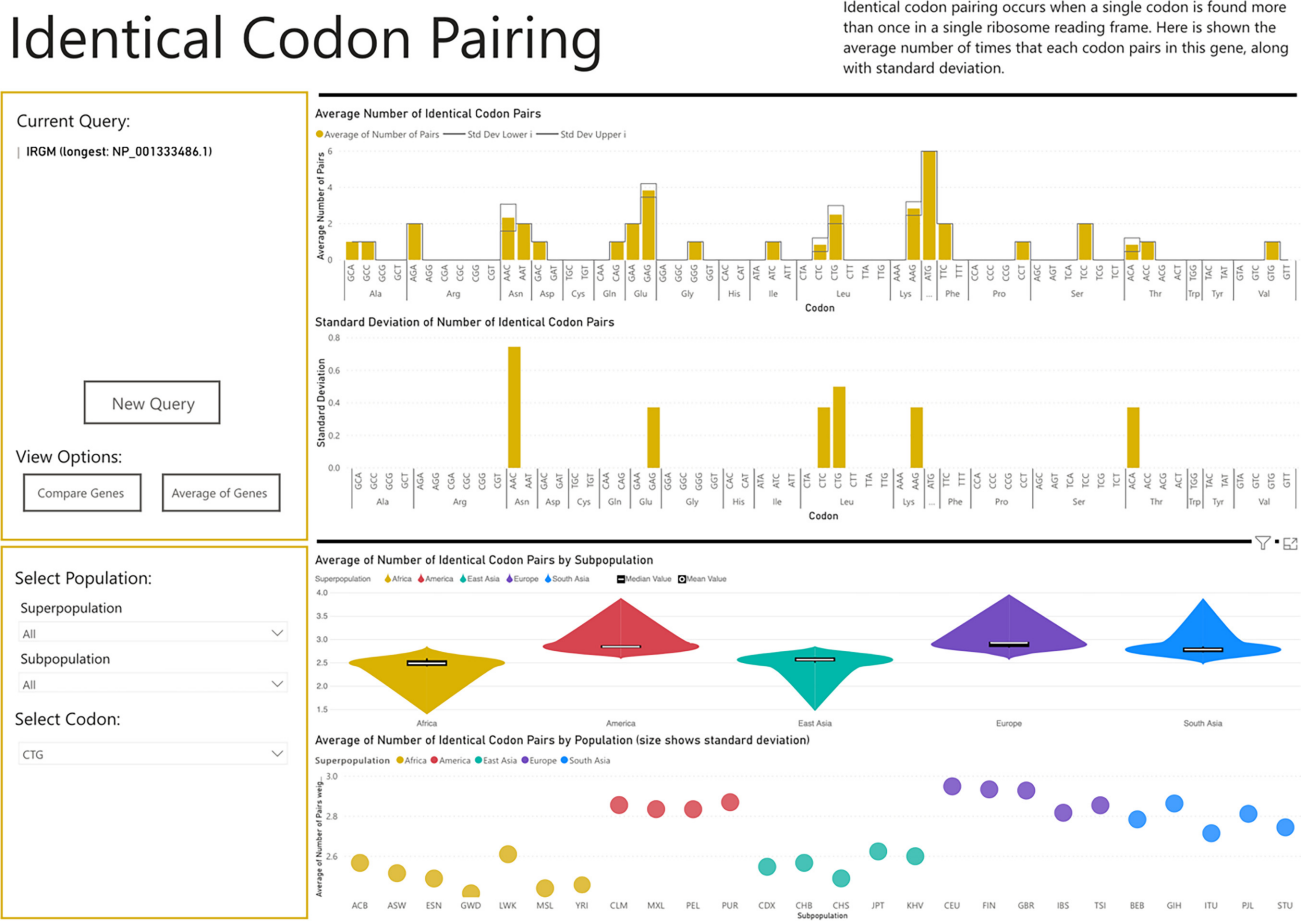
Identical Codon Pairing Screenshot of *IRGM*. A screenshot of the Identical Codon Pairing visual with the *IRGM* gene selected. The lower graphs show population differences in the frequency of CTG pairing, which is decreased by *rs10065172*.

Both African and East Asian populations exhibit a significantly higher MAF of *rs10065172* (MAF = 0.502 and MAF = 0.434 respectively), while the European population has the lowest (MAF = 0.105). Despite not altering the amino acid sequence, *rs10065172* removes a CTG pair, which may alter the efficiency of translation and mRNA secondary structure. Using CUBAP, we found that CTG pairing in *IRGM* varies significantly between populations (see Figure 1). When considering only individuals without synonymous variant *rs10065172*, East Asian and African populations having fewer instances of CTG codon pairing (*P*-value = 2.52 × 10^−41^) than all other populations.

## DISCUSSION

CUBAP enables users to easily visualize cross-population analyses of codon frequencies, codon aversion, identical codon pairing, co-tRNA codon pairing, ramp sequence, and nucleotide composition, including GC content for 17 634 genes and 40 643 isoforms. CUBAP can be used to identify gene-specific and population-specific biases at a codon-by-codon level to help users better understand the extent to which synonymous codon usage biases affect a gene or region of interest. We anticipate that this resource will be used to inspect candidate genes identified in genome-wide association studies, determine the extent to which synonymous codon usage biases are fixed in a population, and predict the effects that changing a codon usage bias will have on different populations. Additionally, our population identification analyses show that biases in codon pairing alone are enough to determine the population of origin in East Asian and African populations, indicating that codon usage might be effectively used in genealogy. CUBAP’s large dataset and interactive, dynamic visuals provide users with the most comprehensive view of codon usage biases across human populations to date.

Since codon usage biases play an integral role in regulating translation rates (3,4,11,25,26), gene expression (7), mRNA and protein secondary structure (12), and can be used in phylogenetics (59,71,72), we anticipate that this resource will be widely used for cross-population analyses and gene-specific inquiries. For instance, traditional genetic analyses of complex diseases rarely evaluate the effects of synonymous codon usage biases because they are generally thought to not contribute significantly to disease. However, recent studies have shown that synonymous codon usage alone significantly alters protein levels and can have a causal effect on Alzheimer's disease (44), cancer (45,46) and multiple sclerosis (45). CUBAP enables researchers to query synonymous codon usage biases in genes identified in case-control studies to determine the extent to which codon usage biases already exist within various populations. Since the values are pre-computed and users can dynamically subtype the data, the web portal is designed to facilitate creative and personalized analyses of specific genes and populations of interest.

We also propose that population-specific codon usage biases can be used as an additional filter in genome-wide association studies. Adjusting for population stratification in genome-wide association studies is difficult (73,74) because variants that are identified as highly associated with disease risk within one population may not be as highly associated in another population (75). We propose that one mechanism that might affect disease association within different populations is the underlying codon usage biases distinct to those populations. For instance, a variant that introduces a codon that was previously averted in one population may not affect the same codon usage dynamic in a different population. Therefore, additional studies might determine if candidate synonymous variants that alter codon dynamics differently between populations are less likely to be generally associated with disease across all populations. Conversely, future studies may find that changing codon usage biases that are uniform across all populations are more likely to have similar pathogenic effects across all populations.

Understanding population-specific codon usage biases may also impact the results of clinical trials of new drugs because drugs can induce different responses depending on the recipient's ethnicity (76–78). Since codon usage dynamics directly affect protein expression levels, population-specific differences in these biases may directly affect a drug's response or dosage requirement within a specific population. Therefore, CUBAP provides a resource for researchers designing clinical trials to visually determine the extent to which codon usage biases might lead to confounding effects between populations.

### Case study: Crohn's disease and *rs10065172*

Despite being in linkage disequilibrium with several other variants (64–66,70,79), *in vitro* experiments show that *rs10065172* alone promotes Crohn's disease development (70). Altered *IRGM* mRNA structure caused by removing a CTG pair in *IRGM* could significantly affect gene expression because *rs10065172* occurs in the seed region of the *IRGM* mRNA, a short sequence vital for microRNA (miRNA) binding and subsequent gene regulation. *In vitro* data reveals that the presence of *rs10065172* in this seed region prevents the binding of MiR-196 microRNAs to *IRGM* mRNA. Without MiR-196 regulation, *IRGM* is overexpressed and alters xenophagy of gut bacteria, thereby increasing the likelihood of developing Crohn's disease (70).

While *rs10065172* increases Crohn's disease susceptibility in Europeans, it is not strongly associated with disease in Africans and East Asians. Additionally, despite having a significantly higher MAF in individuals of African and East Asian descent, Crohn's disease prevalence is relatively low in these populations, especially in Africa, compared to North America and Europe (80), suggesting other population-specific biases may limit the deleterious effects of *rs10065172*. African and East Asian individuals without *rs10065172* have a significantly lower frequency of CTG pairing than individuals without *rs10065172* in other populations (*P*-value = 2.52 × 10^−41^). Because the translation efficiency or optimality of codons correlates with their frequency, the loss of a CTG pair caused by *rs10065172* may have a more pronounced effect on translation in European populations that have significantly higher average CTG pairing in *IRGM*. Conversely, since baseline CTG pairing in *IRGM* is less common in African and East Asian populations, *rs10065172* may have a more muted effect on translational dynamics, which would explain its lower association with Crohn's disease in those populations. CUBAP provides a platform for analyzing these population-specific codon preferences that provide contextual support to explain a likely mechanism by which *rs10065172* promotes Crohn's disease development in certain populations. The same process can be applied to any variant by using CUBAP to determine the extent to which population-specific codon usage biases affect a specific gene, which may contribute to differential disease association between populations.

Although 35.8% of genes have at least one codon that exhibits a significantly different frequency of codon pairing between populations, each gene averages only 3.044 codons with a significantly different number of pairings between at least two populations. Therefore, only 1.75% of codons have significantly different codon pairings between populations. Similar to the global average, *IRGM* has only three codons that significantly differ between populations: CTG, GAG and ACA. Additionally, the likelihood of correctly choosing at random two out of five superpopulations that were previously implicated as having a decreased risk for Crohn's disease would be 10%. Therefore, the probability of correctly identifying a specific codon pairing as affecting only two populations is represented by 0.0175 × 0.1 = 1.75 × 10^−3^ = 0.175%. Given that the probability of randomly identifying CTG pairing in *IRGM* as affecting Crohn's disease in only African and East Asian populations is very low (*P*-value = 1.75 × 10^−3^), our case study illustrates the utility of CUBAP in adding additional support to previous studies, as well as narrowing the search space for potential impacts of population-specific codon usage biases on disease. Additionally, CUBAP provides a starting point for researchers who may not know the effects of a variant *a priori* to identify codons that have significant population-specific differences in various codon usage biases that are predicted to affect gene expression.

## CONCLUSION

Analytical codon usage bias tools have played an important role in understanding the dynamics and effects of codon usage bias in genes and species. While many codon usage bias calculators and databases exist, CUBAP offers the most comprehensive and interactive analysis of codon usage biases across human populations to date. CUBAP is a powerful tool that allows researchers to easily compare codon frequency, codon aversion, identical codon pairing, co-tRNA codon pairing, ramp sequences, and nucleotide composition across 26 populations and five superpopulations. We anticipate that this tool will allow researchers to better visualize codon usage biases within specific genes, identify population differences in codon usages, and better predict potential implications of synonymous variants associated with genetic diseases.

## Supplementary Material

gkaa863_Supplemental_File
