# Supplementary material for: CUBAP: an interactive web portal for analyzing codon usage biases across populations
Source: Nucleic Acids Res. 2020 Oct 12;48(19):11030–9. doi: 10.1093/nar/gkaa863 (PMC7641757; doi:10.1093/nar/gkaa863)
Supplement: gkaa863_Supplemental_File [file gkaa863_supplemental_file.docx]

**Supplemental Information for CUBAP: An Interactive Web Portal for Analyzing Codon Usage Biases Across Populations**

Matthew W. Hodgman^1†^, Justin B. Miller^1†^, Taylor E. Meurs^1^, and John S.K. Kauwe^1*^

^1^ Department of Biology, Brigham Young University, Provo, Utah, 84602, United States

* Correspondence should be addressed to JSKK. Tel: 801-422-2993; Fax: 801-422-0900; Email: kauwe@byu.edu

^†^The authors wish it to be known that, in their opinion, the first two authors should be regarded as co-first authors.

Table of Contents

[Tables 3](#_Toc48116513)

[Table S1: Bonferroni P-value Thresholds 3](#_Toc48116514)

[Table S2: ANOVA of Population-specific Minor Allele Frequencies of Synonymous Pathogenic Variants in the 1000 Genomes Project 4](#_Toc48116515)

[Table S3: Tukey Test P-values of Population-specific Minor Allele Frequencies of *rs10065172* in the 1000 Genomes Project 5](#_Toc48116516)

[Figures 6](#_Toc48116517)

[Figure S1: Co-tRNA and Identical Codon Pairing 6](#_Toc48116518)

[Figure S2: Ramp Sequences 7](#_Toc48116519)

[Figure S3: Flowchart Depicting Data included in CUBAP 8](#_Toc48116520)

[Figure S4: Codon Frequencies Screenshot 9](#_Toc48116521)

[Figure S5: Identical Codon Pairing Screenshot 10](#_Toc48116522)

[Figure S6: Co-tRNA Codon Pairing Screenshot 11](#_Toc48116523)

[Figure S7: Codon Aversion Screenshot 12](#_Toc48116524)

[Figure S8: Ramp Sequences Screenshot 13](#_Toc48116525)

[Figure S9: Nucleotide Composition Screenshot 14](#_Toc48116526)

# Tables

## Table S1: Bonferroni P-value Thresholds

| Bias Type | Number of ANOVA tests | Corrected α for ANOVA | Additional t-tests of significant ANOVAs | | | |
| --- | --- | --- | --- | --- | --- | --- |
|  |  |  | **Superpopulation**  **number of tests** | **Superpopulation corrected alpha** | **Subpopulation number of tests** | **Subpopulation corrected alpha** |
| Codon Frequency | 1,128,576 | 4.43 x 10^-8^ | 11,285,760 | 4.43 x 10^-9^ | 366,787,200 | 1.36 x 10^-10^ |
| Codon Aversion | 1,128,576 | 4.43 x 10^-8^ | 11,285,760 | 4.43 x 10^-9^ | 366,787,200 | 1.36 x 10^-10^ |
| Identical Pairs | 1,075,674 | 4.64 x 10^-8^ | 10,756,740 | 4.64 x 10^-9^ | 349,594,050 | 1.43 x 10^-10^ |
| Co-tRNA Pairs | 352,680 | 1.42 x 10^-7^ | 3,526,800 | 1.42 x 10^-8^ | 114,621,000 | 4.36 x 10^-10^ |
| Ramp Sequences | 6,021 | 8.30 x 10^-6^ | 60,210 | 8.30 x 10^-7^ | 1,956,825 | 2.56 x 10^-8^ |

The Bonferroni corrected alpha is calculated as 0.05 divided by the total number of tests for each bias type. For codon frequency and aversion, an ANOVA test was performed for every codon in the longest isoform of every gene (64*17,634). Identical pair ANOVAs were calculated for all protein-encoding codons in those isoforms (61*17,634). Co-tRNA pairing ANOVAs were calculated for every amino acid in every longest isoform (20*17,634), and ramp ANOVAs were calculated on the mean harmonic length of every isoform with a ramp sequence (6,021). Each of these tests was then followed with pairwise t-tests (10 tests per ANOVA for superpopulation comparisons, 325 tests per ANOVA for subpopulations).

## Table S2: ANOVA of Population-specific Minor Allele Frequencies of Synonymous Pathogenic Variants in the 1000 Genomes Project

| Variant | P-Value |
| --- | --- |
| *rs190057175* | 0.400713 |
| *rs201728948* | 0.400713 |
| *rs104893837* | 0.182205 |
| *rs10065172* | 1.06x10^-10^ |
| *rs121918138* | 0.243681 |
| *rs104886478* | 0.400713 |
| *rs121918236* | 0.400713 |
| *rs116928232* | 0.615111 |
| *rs201550531* | 0.400713 |
| *rs574462207* | 0.243681 |
| *rs200092283* | 0.400713 |
| *rs143523371* | 0.400713 |
| *rs80338735* | 0.400713 |
| *rs143740376* | 0.243681 |
| *rs149266909* | 0.014296 |

P-values for the 1000 Genomes Project for pathogenic synonymous variants are shown. Many variants had low minor allele frequencies (e.g., only one individual had the genetic variant), which could limit the variant being reported as significant even if it affects a population-specific codon usage bias.

## Table S3: Tukey Test P-values of Population-specific Minor Allele Frequencies of *rs10065172* in the 1000 Genomes Project

| **Comparison** | **Adjusted P-Value** |
| --- | --- |
| **Africa-Europe** | 3.42x10^-8^ |
| **Africa-America** | 2.60x10^-6^ |
| **East Asia-Europe** | 3.06x10^-6^ |
| **Africa-South Asia** | 7.27x10^-6^ |
| **East Asia-America** | 2.28x10^-4^ |
| **East Asia-South Asia** | 6.09x10^-4^ |
| **South Asia-Europe** | 0.120906 |
| **America-Europe** | 0.264869 |
| **Africa-East Asia** | 0.763727 |
| **South Asia-America** | 0.990709 |

Tukey test p-values of population-specific difference in the minor allele frequencies of *rs10065172* in the 1000 Genomes Project. Africa and East Asia have significantly different minor allele frequencies for *rs10065172* from every other superpopulation except each other. No other comparisons were significant.

# Figures

## Figure S1: Co-tRNA and Identical Codon Pairing


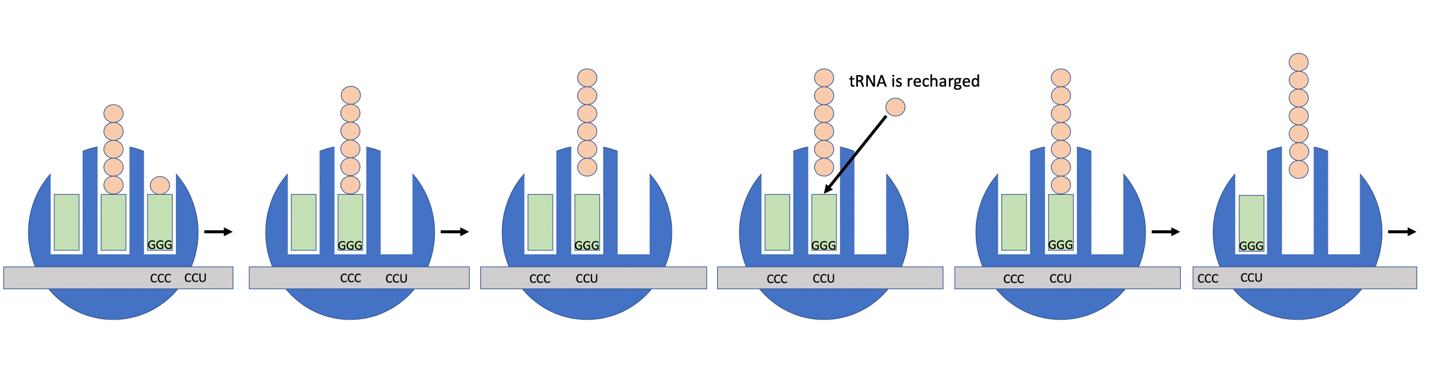


Co-tRNA codon pairing occurs when synonymous, but not identical, codons are present in the same ribosome reading frame. Likewise, identical codon pairing occurs when identical codons are present in the same ribosome window. The proximity of the codons allows the tRNA used on the first codon to be recharged before it diffuses from the ribosome and then used again on the second codon. This mechanism increases overall translational efficiency because the ribosome does not need to wait for another aminoacyl-tRNA to arrive before continuing translation. The ribosome is illustrated above in blue, with the mRNA strand in grey. The tRNA are shown as green rectangles and the amino acid is a beige circle. When two codons that encode the same amino acid (e.g., CCU and CCC) appear within the ribosome simultaneously, co-tRNA codon pairing would allow the tRNA (e.g., GGG) to be recharged with an amino acid and translate both codons without diffusing from the ribosome.

## Figure S2: Ramp Sequences

A ramp sequence is a short segment of slowly translated codons near the beginning of a gene. Codon translational efficiency can be measured using the Relative Synonymous Codon Usage (RSCU) value. Ramp sequences are identified by calculating the harmonic mean of the RSCU values of the codons within a ribosomal window (e.g., nine codons). Lower RSCU values generally indicate less efficient translation. However, the presence of a ramp of relatively low RSCU values relative to the rest of the gene sequence concentrated at the beginning of the sequence allows ribosomes to evenly space and prevent "traffic jams" downstream during translation, effectively increasing overall translational efficiency. The ramp sequence from the gene *UNKL (NP_001032202.1)* is highlighted in orange above. This 20-codon long segment has a harmonic mean RSCU value of 0.4531 while the entire gene has a harmonic mean RSCU value of 0.7734.

##

## Figure S3: Flowchart Depicting Data included in CUBAP


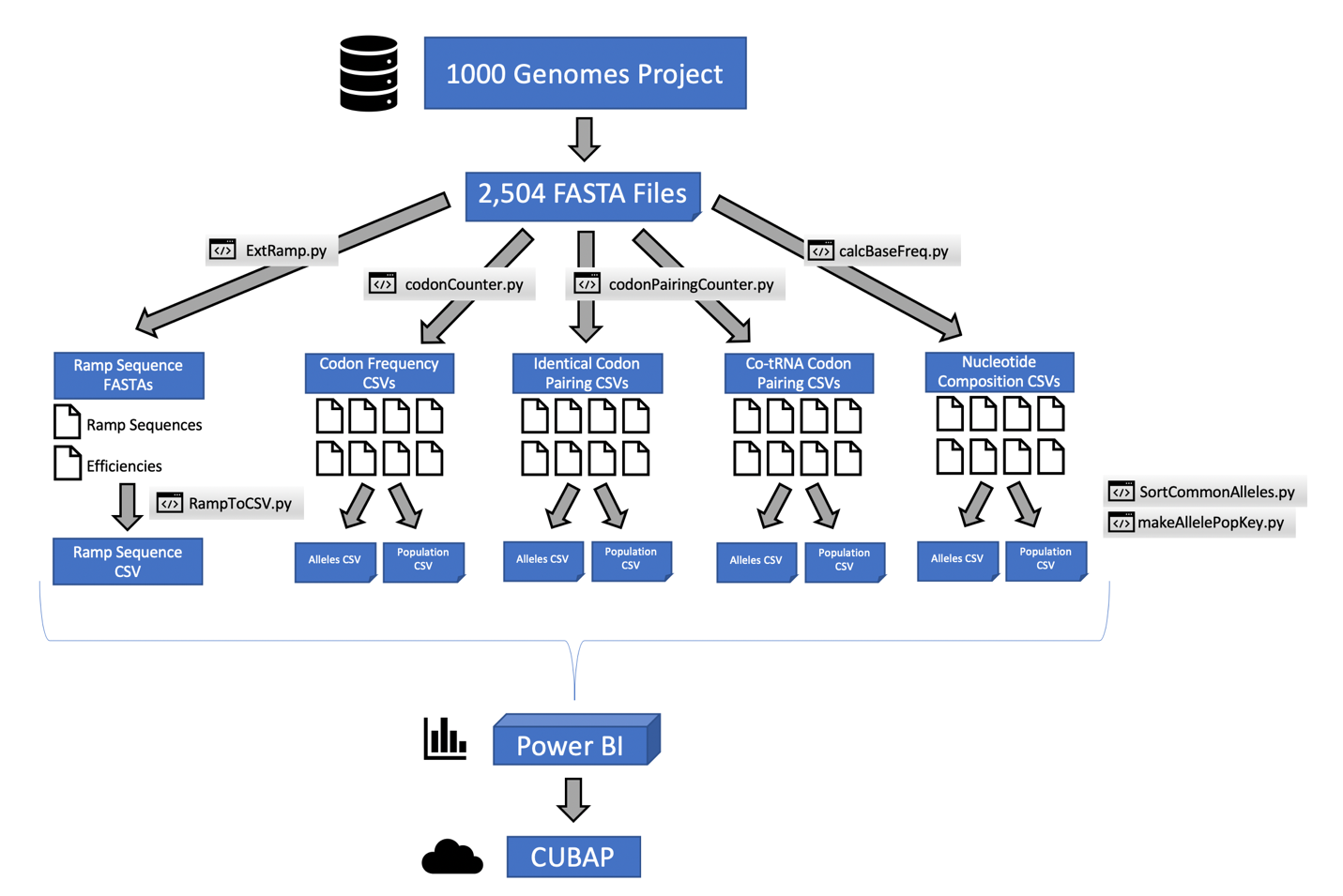


FASTA files from the 1000 Genomes Project were run through several different Python scripts to calculate codon usage bias data and incorporate them into Power BI for visualization on CUBAP. Codon frequency comma separated values (CSV) files have the following headers: sample name, gene name, isoform name, and frequency of each codon. Identical codon pairing and co-tRNA codon pairing data were written to separate CSV files containing the sample name, gene name, isoform name, and the frequencies of each pair. Ramp sequences were written to a CSV file including the sample name, subpopulation, superpopulation, gene name, harmonic mean of the relative synonymous codon usage in the gene, harmonic mean of the relative synonymous codon usage in the ramp, gene length, and ramp length. Nucleotide composition was stored in a CSV file that contained the following columns: sample name, gene name, isoform name, and the frequency of A, T, G, and C. All CSVs are freely available on the CUBAP website.

## Figure S4: Codon Frequencies Screenshot


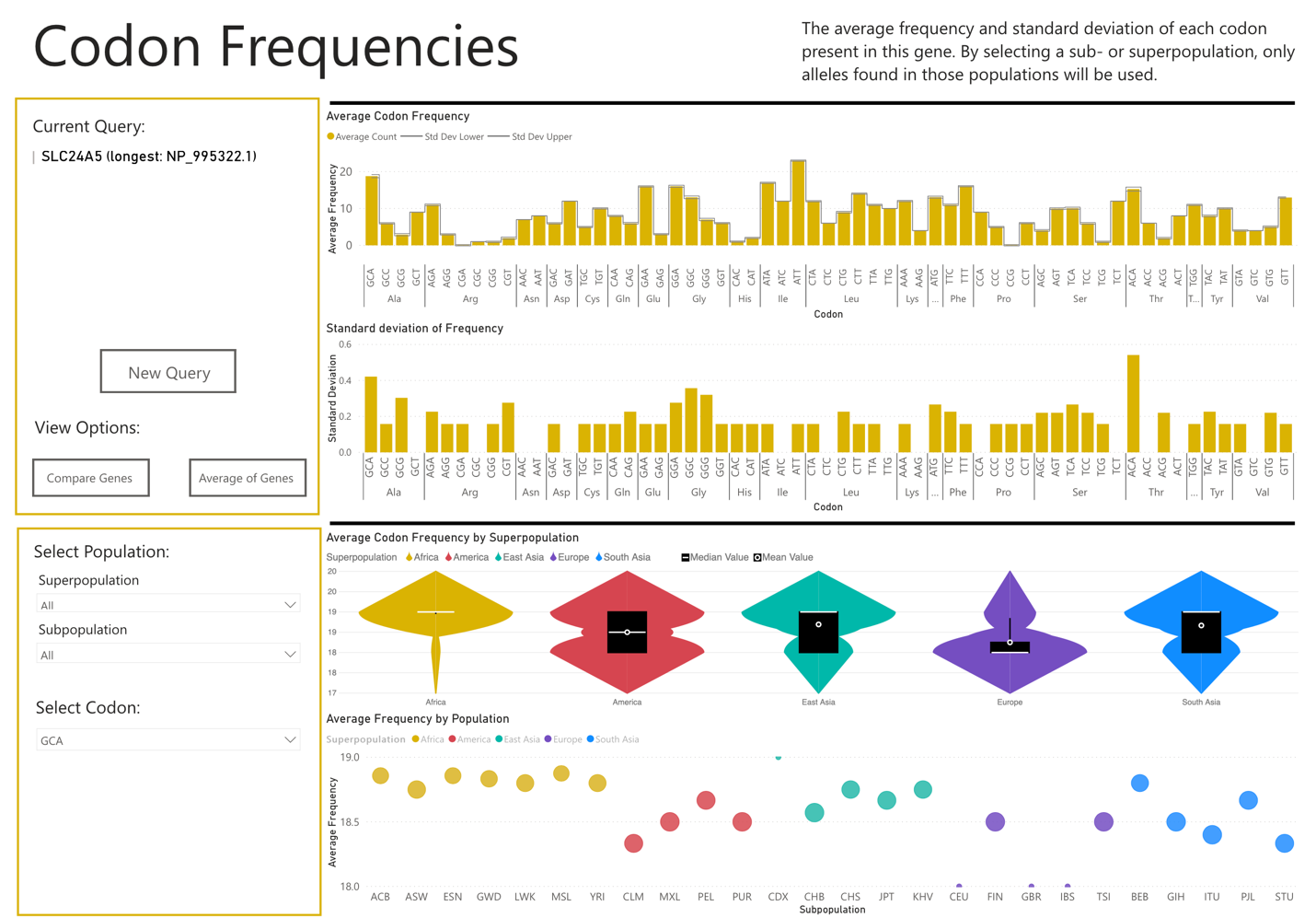


A screenshot of the Codon Frequencies visual. Here, the gene *SLC24A5* is selected. The average and standard deviations of codon frequencies across populations are shown. For the bottom two graphs, the codon 'GCA' has been selected and its frequency across populations can be compared.

## Figure S5: Identical Codon Pairing Screenshot


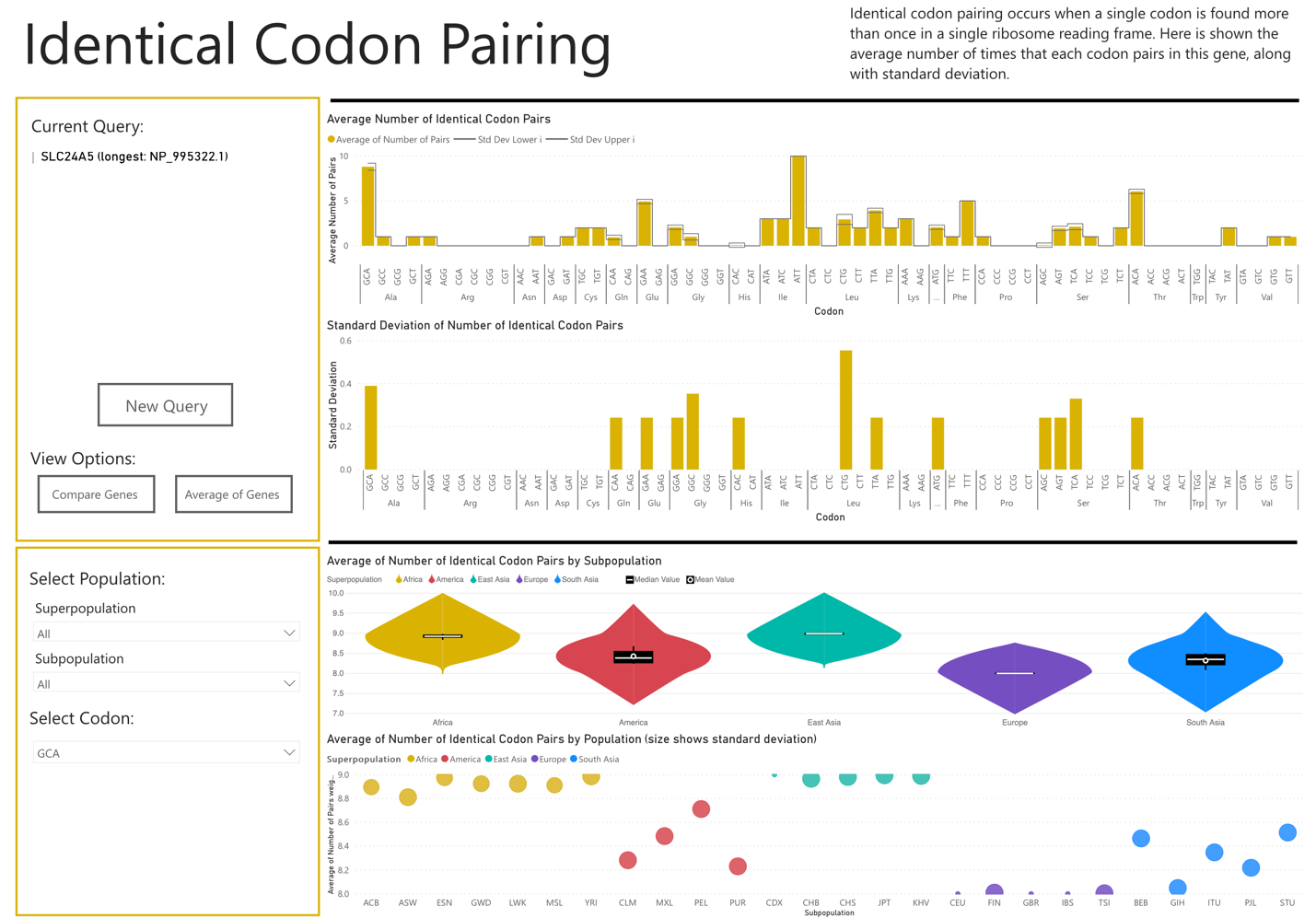


A screenshot of the Identical Codon Pairing visual with the gene *SLC24A5* selected from the website. The lower two graphs show population differences in the frequency of the currently selected codon 'GCA.'

## Figure S6: Co-tRNA Codon Pairing Screenshot


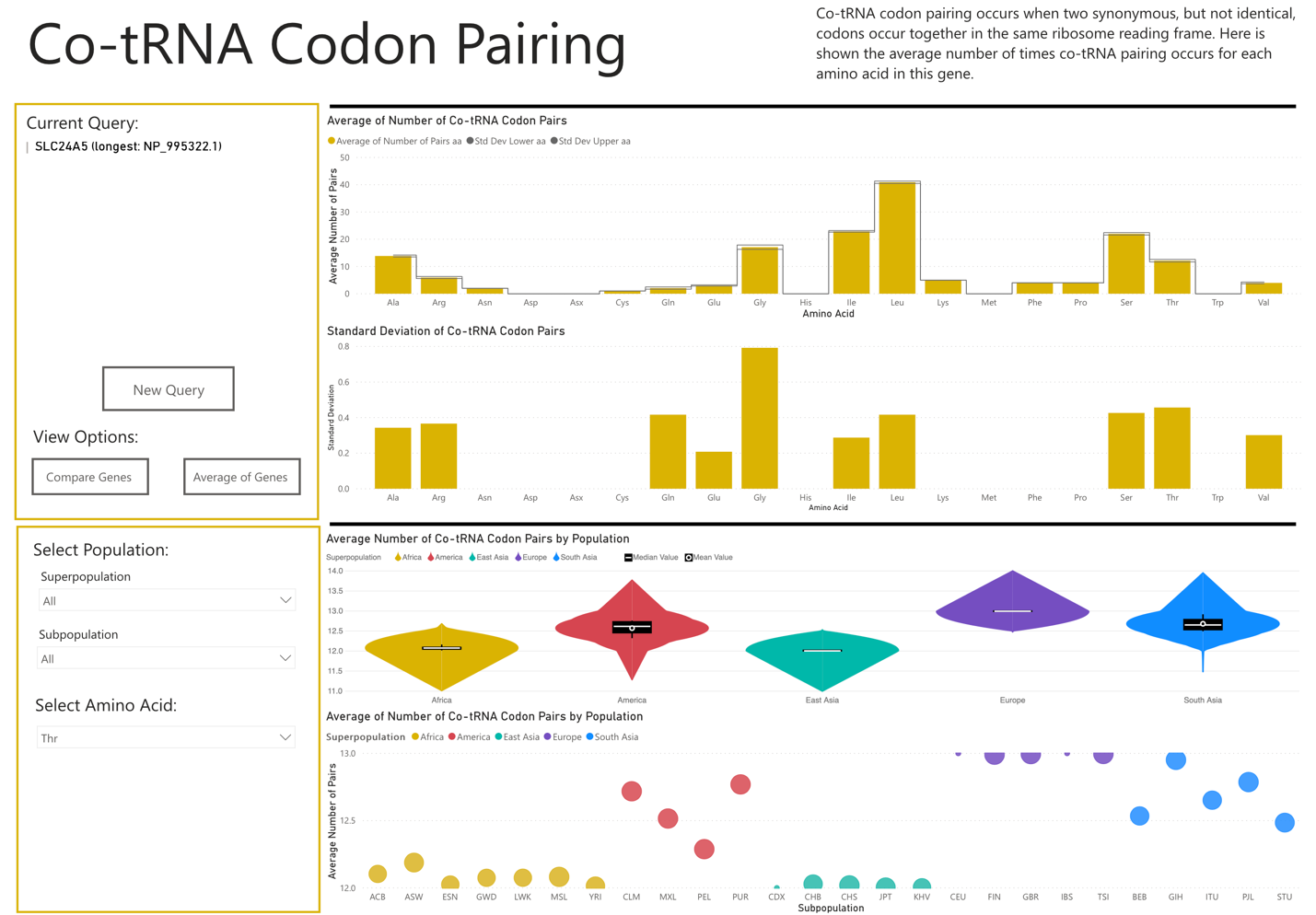


A screenshot of the Co-tRNA Codon Pairing visual with the *SLC24A5* gene selected. The lower graphs show population differences in the frequency of pairing of codons that encode threonine (Thr).

## Figure S7: Codon Aversion Screenshot


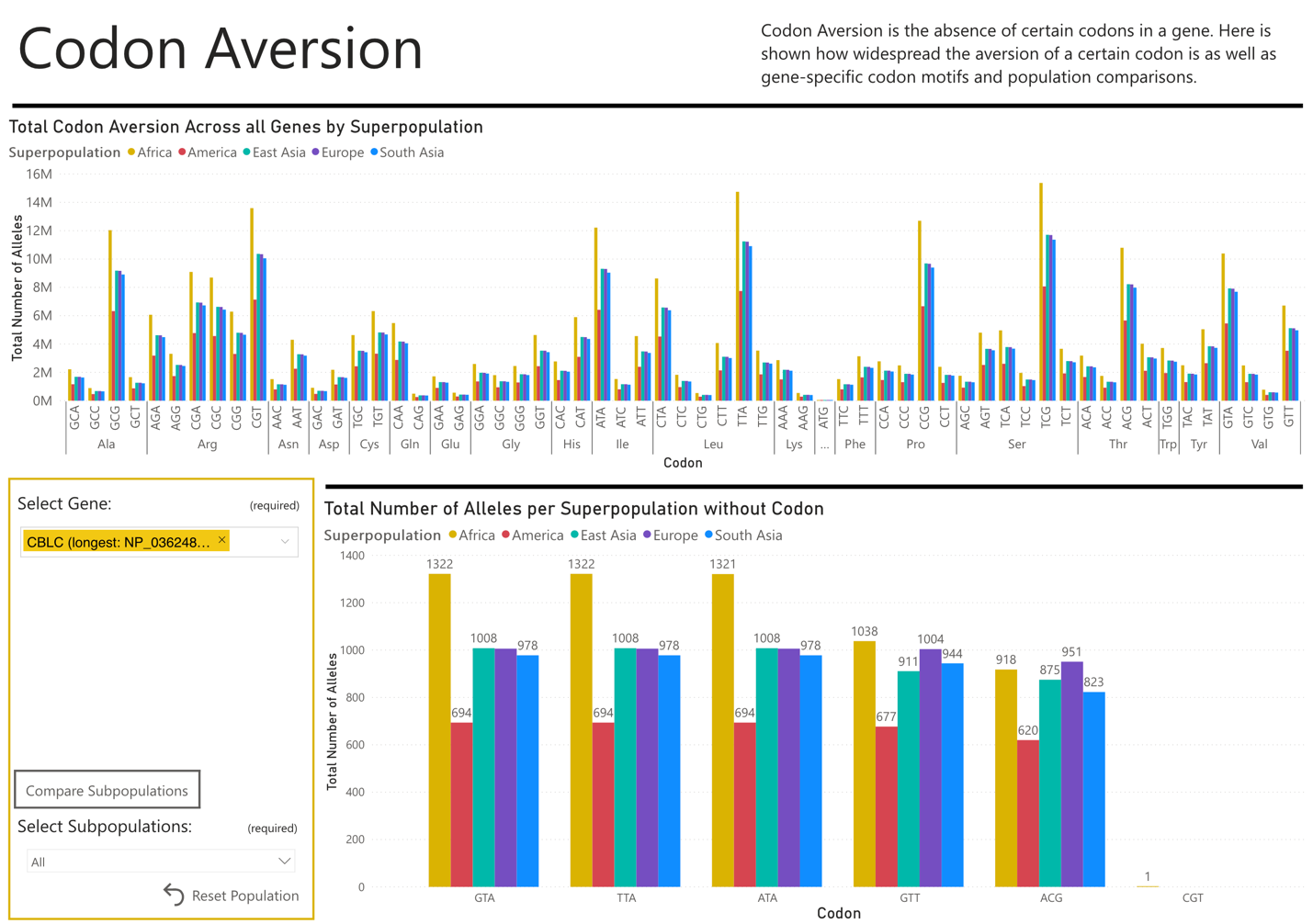


A screenshot of the Codon Aversion visual. The top graph shows codon aversion across all genes. The bottom graph shows the number of individuals that avert codons in the selected *CBLC* gene.

## Figure S8: Ramp Sequences Screenshot


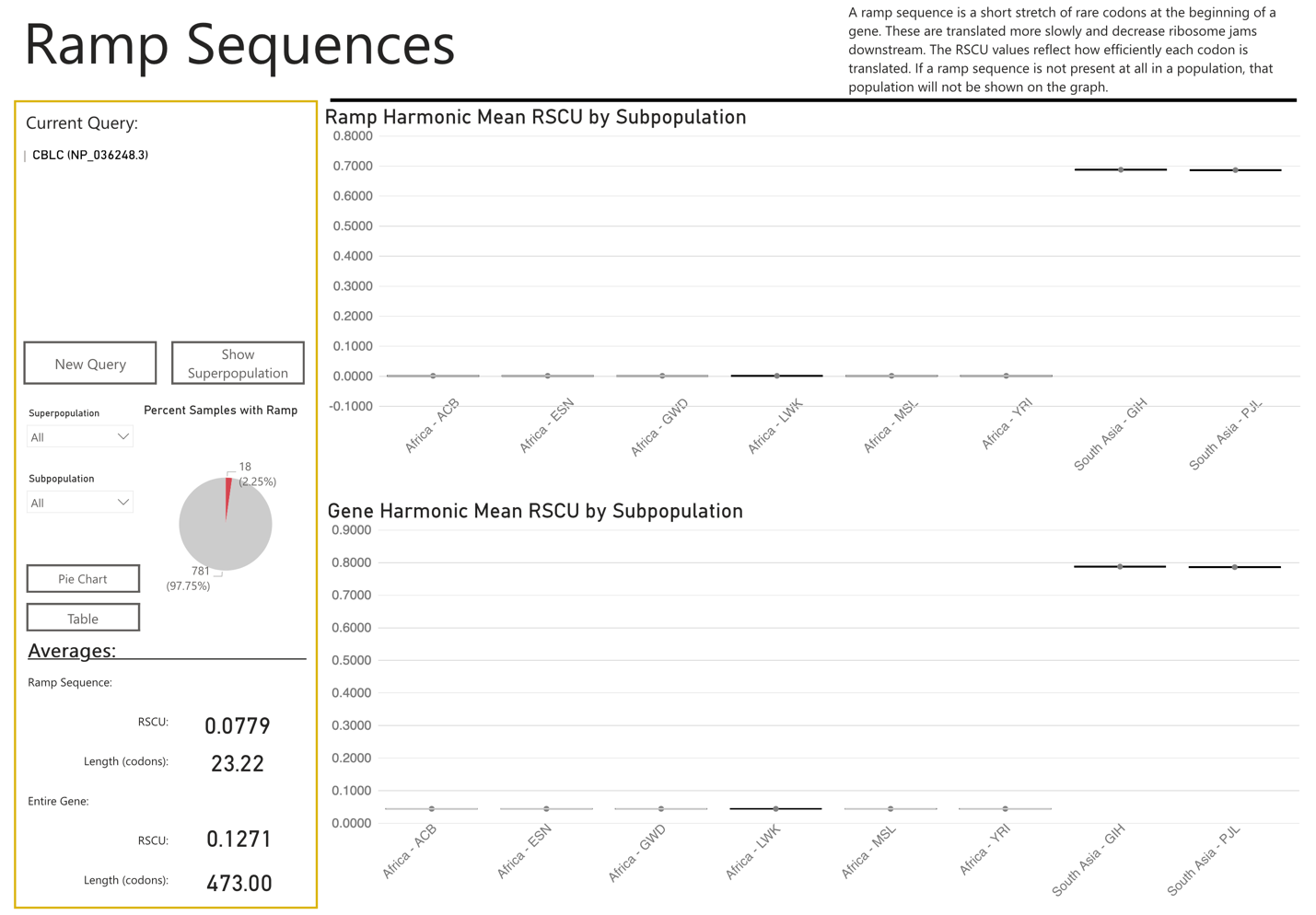


A screenshot of the Ramp Sequence visual. Shown are the relative synonymous codon usage values of codons in the *CBLC* gene. Only some individuals in the African and South Asian populations have a ramp sequence in this gene.

## Figure S9: Nucleotide Composition Screenshot


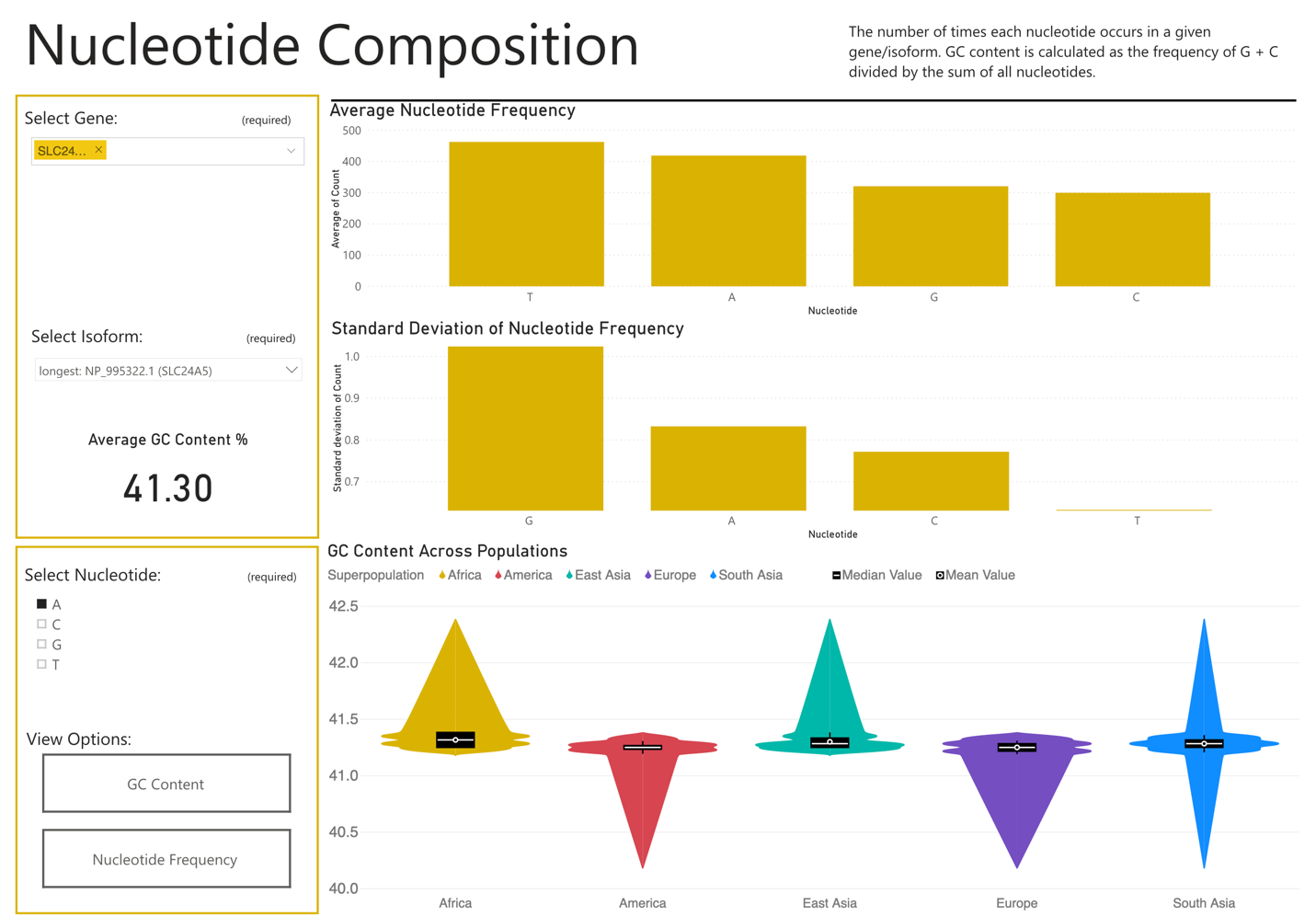


A screenshot of the Nucleotide Composition visual. The *SLC24A5* gene is selected, showing the frequency of each nucleotide as well as population differences in GC content.
